# Book Notices

**Published:** 1867-12

**Authors:** 


### Book Notices.

We have space, in the present number, only sufficient to acknowledge the reception of the following important works:

The Practice of Medicine and Surgery Applied to the Diseases and Accidents Incident to Women. By Wm. H. Byford, A.M., M.D., Author of "A Treatise on the Chronic Inflammation and Displacements of the Unimpregnated Uterus," and Professor of Obstetrics and Diseases of Women and Children in the Chicago Medical College. Second edition, enlarged. Pp. 616. Philadelphia: Lindsay & Blakiston. 1867. Price, $5.00.

Studies in Pathology and Therapeutics. By SAMUEL HENRY DICKSON, M.D., LL.D., Professor of Practice of Physic in Jefferson Medical College, etc., etc. New York: WM. WOOD & Co. Publishers, 61 Walker Street. 1867. Duodecimo. Pp. 201. For sale by W. B. KEEN & Co., 148 Lake Street.

Headaches: Their Causes and their Cure. By HENRY G. WRIGHT, M.D., M.R.C.S.L., L.S.A., Member of Royal College of Physicians of England, etc., etc. From the Fourth London Edition. Duodecimo. Pp. 154. Philadelphia: LINDSAY & BLAKISTON. 1867. Price, $1.25.

Inhalation: Its Therapeutics and Practice. A Treatise on the Inhalation of Gases, Vapors, Nebulized Fluids, and Powders, including a description of the Apparatus employed, and a record of numerous experiments, Physiological and Pathological, with cases. By J. SOLIS COHEN, M.D. Illustrated. Philadelphia: LINDSAY & BLAKISTON. 1867. Price, $2.50. For sale by S. C. GRIGGS & Co., 39 and 41 Lake Street.

Notes on the Origin, Nature, Prevention, and Treatment of Asiatic Cholera. By JOHN C. PETERS, M.D. Second Edition, with Appendix. New York: D. VAN NOSTRAND, 192 Broadway. 1867. Duodecimo, pp. 200.

Epidemic Meningitis, or Cerebro-Spinal Meningitis. By ALFRED STILLE, M.D., Prof. of Theory and Practice of Medicine, and of Clinical Medicine, in the University of Pennsylvania, etc., etc. Philadelphia: LINDSAY & BLAKISTON. 1867. For sale by COBB, PRITCHARD, & Co., 81 Lake St. Price, $2.00.

Hufeland's Art of Prolonging Life. Edited by ERASMUS WILSON, F.R.S., Author of A System of Human Anatomy, etc., etc. Philadelphia: LINDSAY & BLAKISTON. 1867. Pp. 298. Duodecimo. Price, $1.25. For sale by COBB, PRITCHARD, & Co., 81 Lake Street.

Biennial Retrospect of Medicine, Surgery, and Allied Sciences. Edited by Mr. H. POWER, Dr. ANSTIE, Mr. HOLMES, Mr. THOMAS WINDSOR, Dr. BARNES, and Dr. C. HILTON FAGGE, for the New Sydenham Society. Philadelphia: LINDSAY &

BLAKISTON. 1867. For sale by S. C. GRIGGS & Co., 39 and 41 Lake Street. Price, $3.50.

Lectures on Diseases of Women. By CHARLES WEST, M.D., Fellow of the Royal College of Physicians, etc., etc., etc. Third American from the Third and Revised English Edition. Philadelphia: HENRY C. LEA. 1867. For sale by W. B. KEEN & Co. Price, $3.25.

